# [Retracted] Cinobufagin induces autophagy-mediated cell death in human osteosarcoma U2OS cells through the ROS/JNK/p38 signaling pathway

**DOI:** 10.3892/or.2025.8928

**Published:** 2025-06-16

**Authors:** Kun Ma, Chuan Zhang, Man-Yu Huang, Wu-Yin Li, Guo-Qiang Hu

Oncol Rep 36: 90-98, 2016; DOI: 10.3892/or.2016.4782

Following the publication of this paper, it was drawn to the Editor's attention by a concerned reader that certain of the cellular data shown in Fig. 2C on p. 93 had already been submitted to another journal in an article written by different authors at different research institutes, which has subsequently been retracted. Moreover, upon performing an independent assessment of the data in the above paper in the Editorial Office, it emerged that flow cytometric data in Fig. 1 and Fig. 4 were also strikingly similar to data appearing in other unrelated articles, and western blot data appeared to have been shared between Fig. 6A and E on p. 97, where the results from differently performed experiments were intended to have been portrayed.

Owing to the fact that the contentious data in the above article had already been submitted to/published in unrelated articles, and given the fact that Fig. 6 had clearly been assembled incorrectly, the Editor of *Oncology Reports* has decided that this paper should be retracted from the Journal. After having been in contact with the authors, they accepted the decision to retract the paper. The Editor apologizes to the readership for any inconvenience caused.

